# A Catechol-Meter Based on Conventional Personal Glucose Meter for Portable Detection of Tyrosinase and Sodium Benzoate

**DOI:** 10.3390/bios12121084

**Published:** 2022-11-27

**Authors:** Tao Tian, Wei-Yi Zhang, Hang-Yu Zhou, Li-Jing Peng, Xi Zhou, Hao Zhang, Feng-Qing Yang

**Affiliations:** 1 Chongqing Key Laboratory of High Active Traditional Chinese Drug Delivery System, Chongqing Medical and Pharmaceutical College, Chongqing 401331, China; 202018021153t@cqu.edu.cn; 2 School of Chemistry and Chemical Engineering, Chongqing University, Chongqing 401331, China; 202018021117@cqu.edu.cn (W.-Y.Z.); 202018021118@cqu.edu.cn (H.-Y.Z.); 202018021124@cqu.edu.cn (L.-J.P.); 202018021119@cqu.edu.cn (X.Z.); fengqingyang@cqu.edu.cn (F.-Q.Y.)

**Keywords:** tyrosinase, sodium benzoate, personal glucose meter, enzyme sensor, carbonate beverages sample

## Abstract

In this study, the personal glucose meter (PGM) was first used as a fast and user-friendly meter for analyzing catechol (CA) based on the reduction of the mediator K_3_[Fe(CN)_6_] to K_4_[Fe(CN)_6_] in the glucose test strip. Then, an easy, low-cost, and convenient PGM-based method for detecting tyrosinase (TYR) activity and sodium benzoate (SBA) was developed on the basis of the TYR-catalyzed reaction. In this method, CA is oxidized to form *o*-benzoquinone by TYR, thereby reducing the residual amount of CA and the PGM readout. On the other hand, SBA can inhibit the oxidation of CA catalyzed by TYR and increase the residual amount of CA after the enzymatic reaction. Therefore, the activity of TYR is proportional to the difference in the PGM readout of CA, and the concentration of SBA is positively correlated with the residual amount of CA. After the relevant experimental conditions were systematically optimized, the proposed PGM-based method for the detection of TYR and SBA was successfully validated. The liner ranges are 1.0–103.3 U/mL and 6.25–1000 ppm, and the quantification limits are 1.0 U/mL and 6.25 ppm for TYR and SBA, respectively. Moreover, the spiked recovery tests in normal human serum and carbonate beverages (i.e., Cola, Sprite, and Fanta) were performed, and the recoveries (91.6–106.8%) further confirm the applicability of the PGM-based method in real sample analysis.

## 1. Introduction

Benzoic acid (BA) and its salts are broad-spectrum antimicrobial agents that can effectively prevent most bacterial attacks, as well as control mold and inhibit yeast growth [1]. Therefore, they are often used as a preservative in carbonated beverages, soy sauce, preserved fruit, and functional drinks to extend their shelf life [2]. Among them, sodium benzoate (SBA) is one of the most frequently used acid-type food preservatives, which is converted to the active form BA upon use [3]. Nowadays, SBA is more commonly used than BA due to its better water solubility and stability [4]. However, the levels of BA and its salts higher than the maximum allowable value can cause some adverse effects, such as disturbing the plasma acid-base balance, triggering allergic reactions, causing liver damage and skin diseases, as well as leading to convulsions and asthma [5,6,7]. In non-alcoholic carbonated beverages, SBA may react with vitamin C to form benzene, which is one of the first-class carcinogens [8]. Therefore, the maximum concentration of BA and its salts in foods are strictly controlled by China Food and Drug Administration (CFDA). For example, the concentration of SBA in preserved fruit should not exceed 0.5 g/kg, fruit-vegetable juice beverages, soy sauce, vinegar, coffee, and tea should not exceed 1.0 g/kg, and carbonated beverages and functional drinks should not exceed 0.2 g/kg [9].

Several traditional methods have been reported for the determination of SBA, including capillary electrophoresis [10,11], high performance liquid chromatography (HPLC) [12,13,14,15], and spectrophotometry [16,17]. Although most of these methods have high accuracy and reliability, they usually require expensive and bulky instruments, professional operators and complex sample pretreatments. These shortcomings limit the applications of these methods in on-site analysis. Point-of-care testing (POCT) is a portable, inexpensive, and easy-to-use technique that can quickly obtain results and realize instant on-site detection of analytes [18]. As a typical POCT method, the paper-based method has attracted much attention because it can not only be observed directly by the naked eyes, but also requires inexpensive materials. Liu et al. designed a microfluidic paper-based analytical platform based on the Janovsky reaction for the colorimetric detection of BA with concentrations in the range of 500–4000 ppm [19]. Subsequently, Ko et al. developed a microfluidic colorimetric paper-based system combined with three-primary colors (red, green, and blue) analysis to determine SBA in common foods and beverages within 20 min with the detection limit of 50 ppm and concentrations ranging from 50 ppm to 5000 ppm [20]. Nonetheless, paper-based analytical methods still face the disadvantages of requiring synthetic materials, assisted software, and difficulty in direct obtaining accurate results [21].

The personal glucose meter (PGM) is well-known as one of the most mature POCT devices, and it is widely used to monitor and manage the blood glucose of diabetic patients. The PGM exhibits the characteristics of low cost, high sensitivity, easy to operate, stable electrochemical signal, easy-to-read signal numbers and short analysis time [22]. Generally, most of the commercial PGMs are enzyme biosensors based on the current change, and they are mainly composed of a PGM and a glucose test strip [23]. The PGM depends on the amount of current generated by the reaction between glucose and glucose oxidase or dehydrogenase in the test strip to reflect the concentration of glucose in the blood. Based on the testing principle of PGM, many strategies have been investigated to transform PGM into a common meter for analyzing non-glucose targets such as heavy metal ions, enzymes, mycotoxins, pathogens, and pesticides [24,25,26,27,28,29]. Most of these strategies require combination with glucose-loaded materials or glucose-related enzymes to produce glucose, modified glucose test strips, or join with other technical equipment. In essence, the quantitative detection of target analytes is achieved using glucose as the responsive substance in PGM. Another approach to expanding the application of PGMs is focused on the direct redox reaction of the target with the mediator in the glucose test strip. This approach can directly convert PGM into a non-glucose target meter without any conjugation or modification. For example, a portable and user-friendly PGM-based method was developed (by us) to detect hydrogen peroxide using ascorbate oxidase, and the PGM readout triggered by the ascorbic acid achieved a detection quantification of 2.5 μM for hydrogen peroxide [30]. Zhang et al. developed an easy and green PGM method to detect quantification of hydrogen peroxide and hypochlorite in household disinfectants on the basis of the acetylthiocholine iodide-mediated reaction with the quantification limit of 1.7 mM and 0.9 mM, respectively [31]. As a common mediator in many glucose test strips, potassium ferricyanide (K_3_[Fe(CN)_6_]) is an important element for this conversion, which facilitates the electron transfer between the enzymatic reaction and the electrodes to generate the PGM detectable signal.

**Figure 1:**
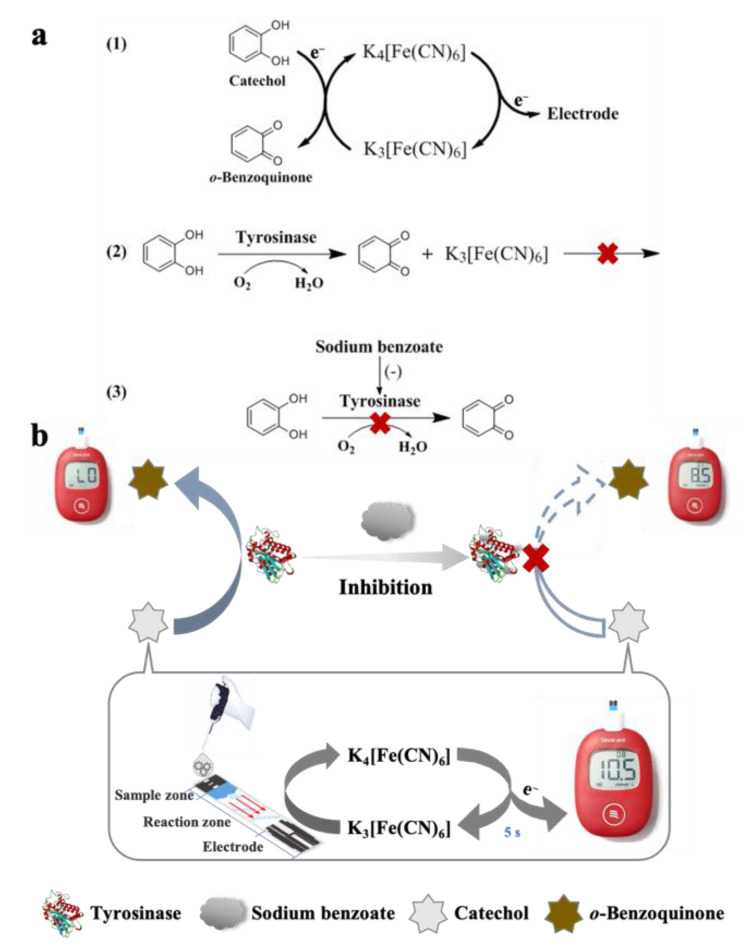
The chemistry of the associated reactions (**a**) and schematic illustration of the detection of TYR activity and SBA based on the PGM signal triggered by CA (**b**). “L0”: no PGM signal or PGM readout <1.1 mM.

Tyrosinase (TYR), also known as polyphenol oxidase, which is a crucial enzyme of melanin synthesis in living organisms, is widely found in microorganisms, animals, plants, and humans [32]. It can catalyze the oxidation of *o*-diphenols to *o*-diquinones in the presence of molecular oxygen [33]. Thus, many electrochemical biosensors have been developed to detect phenolic compounds through immobilizing TYR [34,35]. Moreover, abnormal levels of TYR are closely related to pigmentary disorders, browning of fruits and vegetables, and neurological syndromes such as malignant melanoma and Parkinson’s disease [36,37]. Traditional analysis methods have been developed for TYR activity detection including spectrophotometry [38], fluorometry [38,39], electrochemistry [40], and colorimetry [41]. In these reported studies, tyrosine, as one of the substrates of TYR, can be used to develop biosensors for the detection of TYR activity (Table S1). However, although these methods possess their own advantages, most of these methods require synthetic complex materials, professional operation and high cost, which hinders their widely application in resource-limited areas. In this study, catechol (CA) can react with the mediator K_3_[Fe(CN)_6_] in the glucose test strip to produce K_4_[Fe(CN)_6_] to further generate a PGM detectable readout, thereby converting the PGM into a rapid and user-friendly meter for CA (Figure 1). On the other hand, CA can be oxidized by TYR to *o*-benzoquinone with the change of the solution color from colorless to brown, accompanying the decrease in the amount of CA [42]. Therefore, the analysis of TYR activity can be achieved by monitoring the variation in the PGM readout of CA during the catalyzing process. Furthermore, SBA can inhibit the TYR activity [43]. When SBA is pre-incubated with TYR, the amount of residual CA will be increased accordingly after the same enzymatic reaction. Thus, the SBA can be determined by the PGM based on the inhibition of SBA on TYR without any modification to the PGM and materials. After the systematic optimization of experimental conditions, the spiked recovery tests were performed on the normal human serum and carbonate beverages to confirm the applicability of the proposed method for the detection of TYR activity and SBA, respectively.

## 2. Materials and Methods

### 2.1. Chemicals and Materials

TYR (total enzyme activity of 25 kU) from mushroom, α-glucosidase (α-Glu), alkaline phosphatase (ALP), xanthine oxidase (XOD), glucose oxidase (GOD), ascorbate oxidase (AAO), glutathione (GSH) and D-(+)-glucose were purchased from Shanghai Yuanye Biological Technology Co., Ltd. (Shanghai, China). CA and sucrose were purchased from Aladdin Chemistry Co., Ltd. (Shanghai, China). Sodium hydroxide (NaOH) and sodium dihydrogenorthophosphate (NaH_2_PO_4_) were purchased from Shanghai Titan Scientific Co., Ltd. (Shanghai, China). Fructose, sodium chloride (NaCl), phosphoric acid (H_3_PO_4_) and trisodium citrate dehydrate (SCA) were purchased from Chengdu Chron Chemicals Co., Ltd. (Chengdu, China). SBA and copper sulfate pentahydrate (CuSO_4_·5H_2_O) were purchased from Chuandong Chemical Industry Co., Ltd. (Chongqing, China). KCl was purchased from Beibei Chemical Reagent Factory (Chongqing, China). Calcium chloride anhydrous (CaCl_2_) was purchased from Damao Chemical Reagent Factory (Tianjin, China). Iron (III) chloride (FeCl_3_) was purchased from Beijing MREDA Technology Co., Ltd. (Beijing, China). K_4_[Fe(CN)_6_] was purchased from Shanghai Macklin Biochemical Co., Ltd. (Shanghai, China). K_3_[Fe(CN)_6_] and maltose were purchased from Adamas-beta Reagent Co., Ltd. (Shanghai, China). L-histidine (His) was purchased from Chengdu Huaxia Chemical Reagent Co., Ltd. (Chengdu, China). L-tryptophan (Try) was purchased from Beijing Dingguo Biotechnology Co. Ltd. (Beijing, China). Bovine serum albumin (BSA) was purchased from Sangon Biotech Co., Ltd. (Shanghai, China). Caffeine (>98%) was purchased from Chengdu Biopurify Phytochemicals Ltd. (Chengdu, China). Mannose was purchased from Shanghai regal Biology Technology Co, Ltd. (Shanghai, China). All the solvents used in the HPLC analysis such as methanol were of HPLC-grade and purchased from Adamas-beta Reagent Co., Ltd. (Shanghai, China).

The normal human serum was purchased from Beijing Solarbio Science and Technology Co., Ltd. (Beijing, China). Cola, Sprite, and Fanta were purchased from Colorful Supermarket at Chongqing University (Chongqing, China). Before analysis, these samples were respectively diluted to fit the linear range of calibration plots of the proposed method by 10.0 mM PBS buffer (pH = 6.0).

### 2.2. Instrumentation

Fourier-transform infrared (FT-IR) spectra of K_3_[Fe(CN)_6_], CA, K_4_[Fe(CN)_6_], and the mixture of CA and K_3_[Fe(CN)_6_] were recorded using a Nicolet 550 II (Thermo Scientific Inc., Waltham, MA, USA). The Safe-AQ Smart PGM and blood glucose test strips (measurement range 1.1–33.3 mM) were purchased from Sinocare Inc. (Changsha, China). The pH of solution was measured by a FE28-standard pH meter (Mettler-Toledo Instruments, Shanghai, China). The UC-2H ultrasonic cleaner was purchased from Shanghai Titan Scientific Co., Ltd. (Shanghai, China). The temperature of reaction was maintained by an electric-heated thermostatic water bath (HWS-12, Shanghai Blue-pard Instruments Co., Ltd., Shanghai, China).

### 2.3. Reagent Preparation

Phosphate buffer solution (PBS) was prepared in deionized water containing NaH_2_PO_4_ (10.0 mM) and its pH was changed by the concentrated H_3_PO_4_ and 1.0 M of NaOH. TYR (310.0 U/mL) was prepared by dissolving 0.5 mg TYR in 2.0 mL of PBS buffer (10.0 mM, pH = 6.0). Fresh CA (15.0 mM, pH = 6.0) solution was prepared by dissolving 1.7 mg CA in 1.0 mL of 10.0 mM of PBS buffer (pH = 6.0). Various concentrations of SBA were prepared by 10.0 mM of PBS (pH = 6.0). All interference solutions of different concentrations were prepared by dissolving them in 10.0 mM of PBS (pH = 6.0), respectively. All the solutions were shielded from light before use.

### 2.4. Detection of TYR Activity and SBA by the PGM-Based Method

First, different concentrations of CA solution were directly measured by the PGM as simple as measuring the concentration of blood glucose. In brief, 1.0 μL of CA solution was mixed with 2.0 μL of PBS (10.0 mM, pH = 6.0) and then measured by the PGM. For the detection of TYR activity, the mixture solution was reacted in 3.0 μL of PBS (10.0 mM, pH = 6.0) containing 24.0 mM CA and various concentrations of TYR (1.0–103.3 U/mL) at 25 °C for 15.0 min. After the TYR-catalyzed reaction, the concentration of residual CA in the mixture was detected by the PGM. The variation in the PGM readout of CA before and after the enzymatic reaction was recorded as “∆PGM readout” to reflect the TYR activity in the sample.

For determining the concentration of SBA, 1.0 μL of CA (18.0 mM) solution was mixed with 2.0 μL of PBS (10.0 mM, pH = 6.0), and then 1.0 µL of the mixture solution was measured by the PGM for the readout of fresh CA. Then, the TYR-catalyzed reaction was carried out in 1.0 μL of TYR (52.75 U/mL), 1.0 μL of PBS (10.0 mM, pH = 6.0) and 1.0 μL of CA (18.0 mM) solution. After incubation for 15.0 min at 25 °C, 1.0 µL of the mixture solution was measured by the PGM for the readout of residual CA. Subsequently,1.0 μL of TYR (52.75 U/mL) was pre-incubated with 1.0 μL of different concentrations of SBA (6.25–1000 ppm) at 25 °C for 3.0 min, and then 1.0 μL of CA (18.0 mM) solution was added to trigger the enzymatic reaction and incubated at 25 °C for 15.0 min, then 1.0 µL of the mixture solution was measured by the PGM for the readout of residual CA after the inhibition of TYR by SBA. Finally, the inhibition percentage calculated with Equation (1) was used for evaluation of the inhibitory activity of SBA on TYR and quantification of the concentration of SBA.

### 2.5. Inhibition Kinetics Study and Validation of the PGM-Based Method

The determination of SBA was carried out through its inhibitory effect on TYR. The inhibition percentage *I*(%) can be computed by comparing the PGM readout before and after addition of SBA according to the Equation (1).

(1) $$I\left(\%\right)=\frac{I_{t}-I_{0}}{I_{b}-I_{0}}\times 100$$

where *I_t_* and *I*_0_ are the PGM readouts of residual CA after the TYR-catalyzed reaction in the presence and absence of SBA, respectively. *I_b_* presents the PGM readout of fresh CA solution.

The *Z′* factor is an important parameter to estimate the performance of an analysis strategy, which is calculated by Equation (2).

(2) $$Z^{\prime }=1-\frac{3\sigma_{s}+3\sigma_{c}}{\left|\mu_{s}-\mu_{c}\right|}$$

where *μ_s_* and *μ_c_* represent the average of the PGM readouts of standard (*s*) (without inhibition) and the negative (*c*) control (100% inhibition by the SBA), respectively. The *σ_s_* and *σ_c_* are the standard deviations of the PGM readout. When the inhibition percentage is 100%, *μ_c_* = 0 and Equation (2) can be simplified to Equation (3). When the value of *Z′* factor is greater than 0.5, the proposed method is thought to be precise and robust.

(3) $$Z^{\prime }=1-\frac{3\sigma_{s}}{\mu_{s}}$$

### 2.6. Selectivity and Interferences Studies

For exploring the selectivity and interferences of the PGM-based method, several possible interferences for the detection of TYR activity, including metal ions (1.0 mM of Na^+^, K^+^, Ca^2+^, Cu^2+^, and Fe^3+^), biological small molecules (0.5 mM of glucose, 1.0 mM of Try, His, and GSH), enzymes (245.7 U/mL of α-GLU, 88.3 U/mL of GOD, 87.7 U/mL of AAO, 100.0 U/mL of ALP and XOD), and proteins (0.33 mg/mL of BSA), were investigated. In brief, 1.0 μL of interferences solution without or with TYR (158.25 U/mL) and 1.0 μL of PBS (10.0 mM, pH = 6.0) were incubated with 1.0 μL of CA (24.0 mM) solution for 15.0 min at 25 °C, then, 1.0 μL of the mixture solution was measured by the PGM.

In addition, several possible interferences in the formulas of carbonated beverages (Cola, Sprite, and Fanta) for the detection of SBA by the PGM, including 200 ppm of SCA and caffeine, 2500 ppm of Na_2_HPO_4_, and saccharides (1000 ppm of maltose, sucrose, fructose, mannose, and glucose), were investigated. In brief, 1.0 μL of TYR (158.25 U/mL) was pre-incubated with 1.0 μL of interferences solution without or with SBA for 3.0 min at 25 °C, and then 1.0 μL of CA (18.0 mM) solution was added to trigger the enzymatic reaction and incubated for 15.0 min at 25 °C. Finally, 1.0 μL of the mixture solution was measured by the PGM.

### 2.7. Real Sample Analysis

For exploring the applicability of the PGM-based method, TYR in the normal human serum sample was determined through the standard addition method. The human serum sample was first diluted 100 times using 10.0 mM of PBS buffer (pH = 6.0) before analysis. Different concentrations of TYR (final TYR concentrations of 26.38, 52.75, and 79.13 U/mL) were added into the human serum solution for the spiked recovery tests. In brief, 1.0 μL of human serum sample solution with TYR and 1.0 μL of PBS (10.0 mM, pH = 6.0) were incubated with 1.0 μL of CA (24.0 mM) solution for 15.0 min at 25 °C, then, 1.0 μL of the mixture solution was measured by the PGM.

SBA in the carbonated beverages (Cola, Sprite, and Fanta) were determined through the standard addition method. The three samples were finally diluted 36 times for Cola and 12 times for Sprite and Fanta. Then, different concentrations of SBA (final SBA concentrations of 50, 100, and 200 ppm) were added to the sample solutions for the spiked recovery tests. In brief, 1.0 μL of TYR (158.25 U/mL) was pre-incubated with 1.0 μL of real sample solutions in the present of SBA for 3.0 min at 25 °C, and then 1.0 μL of CA (18.0 mM) was added into the solution and incubated for 15.0 min at 25 °C, and 1.0 μL of the mixture solution was measured by the PGM. In addition, their background readout was measured in the same way by using 1.0 μL of PBS (10.0 mM, pH = 6.0) instead of 1.0 μL of CA (18.0 mM) solution.

### 2.8. HPLC Analysis

The HPLC analysis was performed on an Agilent 1260 series liquid chromatography system (Agilent Technologies, Palo Alto, CA, USA) and controlled by the Agilent ChemStation software. All separations were carried out on an Agilent ZORBAX SB-C18 column (250 × 4.6 mm i.d., 5 µm) and a guard column (ZORBAX SB-C18, 12.5 × 4.6 mm i.d., 5 µm) at a column temperature of 35 °C. The detection wavelength was set at 280 nm and the injection volume of all samples was 5.0 µL. The mobile phase consisting of solvent A (methanol) and solvent B (0.1% formic acid solution) was eluted with the following gradient elution program: 0–3 min, 35% A; 3–9 min, 35%–100% A; 9–12 min, 100%–50% A. The flow rate was at 1.0 mL/min. The samples were filtered through a 0.22 µm membrane filter (Navigator Lab Instrument, Tianjin, China) before being injected into HPLC for analysis.

The SBA was prepared with different concentrations of 6.25–1000 ppm in 10.0 mM PBS (pH = 6.0), and a calibration curve for the determination of SBA by HPLC analysis was established. Then, the spiked recovery tests of the carbonated beverage samples (Cola, Sprite, and Fanta) were performed by the HPLC method after spiking with three different concentrations of SBA (final concentrations of 50, 100, and 200 ppm).

## 3. Results and Discussion

### 3.1. Principle of Portable Detection of TYR Activity and SBA

The principle of portable detection of TYR activity and SBA based on CA enabling the PGM readout is illustrated in Figure 1. (1) The PGM is firstly converted into a CA-meter. After the CA solution is adsorbed into the sample zone by capillarity action and enters the reaction zone, K_3_[Fe(CN)_6_] can oxidize CA to *o*-benzoquinone and get the electrons from CA to produce K_4_[Fe(CN)_6_]. Then, K_4_[Fe(CN)_6_] is re-oxidized on the electrode surface of the glucose test strip to generate the microcurrent signal, the PGM can rapidly detect the microcurrent and transform it to a digital readout. Thus, the quantitative detection of CA can be achieved by the PGM. (2) For the detection of TYR activity, TYR can catalyze the oxidation of CA to *o*-benzoquinone, which cannot reduce K_3_[Fe(CN)_6_] to trigger the following reactions. Therefore, the concentration of CA and the PGM readout decreases after the enzymatic reaction. Based on the consumption of CA, TYR activity can be detected through monitoring the changes of PGM readout before and after the enzymatic reaction. (3) For the quantification of SBA, the residual amount of CA after the enzymatic reaction will be increased comparatively with the less obvious color change of solution due to the inhibition of SBA on the TYR activity. Thus, the determination of SBA can be achieved through calculating the inhibition percentage of SBA on the TYR activity.

**Figure 2:**
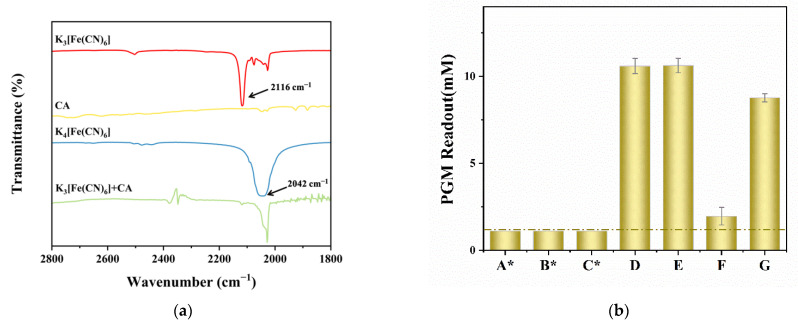
FT-IR spectra of K_3_[Fe(CN)_6_], CA, K_4_[Fe(CN)_6_], and the mixture of K_3_[Fe(CN)_6_] and CA (**a**). The PGM readout of seven kinds of solutions (**b**); A: SBA; B: TYR; C: TYR + SBA; D: SBA + CA; E: CA; F: TYR + CA; G: TYR + SBA + CA; *: no PGM signal or the PGM readout <1.1 mM. Conditions: 103.3 U/mL of TYR, 5.0 mM of CA, 300 and 2000 ppm of SBA, 5.0 min of pre-incubation and incubation time, respectively (*n* = 3). The concentrations mentioned above are final concentrations.

To verify the feasibility and mechanism of PGM for the quantitative detection of CA, the reaction between CA and K_3_[Fe(CN)_6_] was studied by FT-IR analysis. Figure 2a shows the FT-IR spectra of K_3_[Fe(CN)_6_], K_4_[Fe(CN)_6_], CA, and the K_3_[Fe(CN)_6_] after reaction with CA. Two predominant peaks at 2116 cm^−1^ and 2042 cm^−1^ in the FT-IR spectra correspond to the characteristic peaks of K_3_[Fe(CN)_6_] and K_4_[Fe(CN)_6_], respectively [44]. K_3_[Fe(CN)_6_] is easily reduced to K_4_[Fe(CN)_6_] to produce a reversible K_3_[Fe(CN)_6_]/K_4_[Fe(CN)_6_] couple, which facilitates the transference of electrons and the amplification of the microcurrent signal [45]. These two peaks are not observed in the spectrum of CA. After CA was added into the K_3_[Fe(CN)_6_], the characteristic peak of K_3_[Fe(CN)_6_] at 2116 cm^−1^ disappeared, but a new peak corresponding to the K_4_[Fe(CN)_6_] was observed at 2042 cm^−1^. These results demonstrate the conversion of K_3_[Fe(CN)_6_] to K_4_[Fe(CN)_6_], which is also confirmed by the color change of the solution (Figure S1 in Supplementary Materials). After the CA was added, the yellow K_3_[Fe(CN)_6_] solution was obviously faded due to the partial reduction of K_3_[Fe(CN)_6_] to K_4_[Fe(CN)_6_]. Moreover, Figure S2 shows the dose-dependent response of the PGM readout to the CA concentrations (2.0–10.0 mM) with the regression equation of PGM readout = 2.8779 × C_CA_ − 3.3323 (R^2^ = 0.9992). Therefore, the PGM can be directly used as a CA-meter for the quantitative detection of CA. Moreover, the effect of SBA on the PGM readout of CA was studied. Figure S3 shows that after incubation of CA with different concentrations of SBA (0–2000 ppm) for 5.0–15.0 min, the PGM readout of the mixture solution (CA + SBA) had no obvious variation as compared with the CA stock solution. Thus, the presence of SBA has no (or negligible) direct effect on the determination of CA using the PGM.

Furthermore, to evaluate the feasibility of the proposed method for the detection of TYR activity and SBA, seven solutions were measured by the PGM. As shown in Figure 2b, the PGM readout of TYR, SBA, and the mixture solution of TYR + SBA (solution of A, B and C, Figure 2b) are all L0, which mean that there are no PGM response, or the readout is less than 1.1 mM. However, the PGM readout of CA or the mixture solution of CA+ SBA are about 10.6 mM (solution of D or E, Figure 2b). These results further confirm that SBA has no (or negligible) direct impact on the PGM determination of CA even at high concentrations of SBA (2000 ppm). The PGM readout of the mixture solution of TYR + CA after incubation at 25 °C for 5.0 min is about 2.0 mM, and the color of the mixture solution becomes brown (solution of F, Figure 2b). It means that the CA is catalyzed to form *o*-benzoquinone after the enzymatic reaction, and the concentration of residual CA is decreased, resulting in the decrease of PGM readout. Moreover, when SBA (300 ppm) is pre-incubated with TYR for 5.0 min before the addition of CA to initiate the enzymatic reaction, the PGM readout of the mixture solution of TYR + SBA + CA reaches to 8.8 mM, and the color of the solution has exhibited little change compared with that of solution F (solution of G, Figure 2b). It means that SBA can inhibit the oxidation of CA by TYR, resulting in a higher residual CA concentration and PGM readout than that of without SBA. These results show that the proposed method is feasible to detect TYR activity or SBA through monitoring the change in PGM readout.

### 3.2. Optimization of the Experimental Conditions

To achieve a high efficiency for monitoring TYR activity by the developed method, the effects of experimental parameters on the ΔPGM readout, including pH values of buffer solution, incubation temperature, concentration of CA, and incubation time, were investigated.

**Figure 3:**
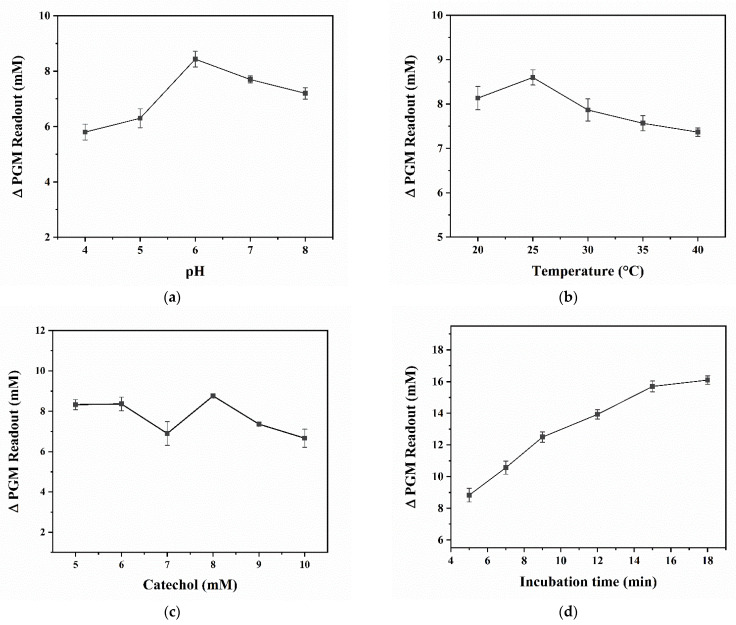
Effects of pH (**a**), temperature (**b**), concentration of CA (**c**), and incubation time (**d**) on the ΔPGM readout. Conditions: 103.3 U/mL of TYR, 5.0 mM of CA, incubation at 20 °C for 5.0 min (**a**); 103.3 U/mL of TYR, 5.0 mM of CA, incubation at 20–40 °C for 5.0 min (**b**); 103.3 U/mL of TYR, 5.0–10.0 mM of CA, incubation at 25 °C for 5.0 min (**c**); 103.3 U/mL of TYR, 8.0 mM of CA, incubation at 25 °C for 5.0–18.0 min (**d**) (*n* = 3). The concentrations mentioned above are final concentrations.

Figure 3a illustrates the variation trend of ∆PGM readout after the enzymatic reaction at different pH values (4.0–8.0). As the pH value increases from 4.0 to 6.0, the ∆PGM readout increases gradually and reaches a maximum value when the pH value is 6.0. Therefore, the pH value of 6.0 was selected for the subsequent experiments, which is consistent with the optimal value provided by the manufacturer. The incubation temperature is an important factor affecting the catalytic activity of enzyme. Figure 3b shows that the ∆PGM readout reached the maximum value at 25 °C and gradually decreased with increasing incubation temperature. Therefore, the incubation temperature of 25 °C was selected for the subsequent experiments. Considering that the liner range of CA detection by the PGM, the effect of final CA concentration in the range of 5.0–10.0 mM on the PGM-based method for TYR activity detection was optimized. As shown in Figure 3c, the ∆PGM readout reaches a maximum value at the final CA concentration of 8.0 mM, which was selected for the subsequent experiments. Moreover, Figure 3d exhibits that the ∆PGM readout is gradually increased with the incubation time of 3.0–18.0 min but leveled off after 15.0 min. The reason can be attributed to the consumption of most of the CA. Therefore, 15.0 min was selected as the optimal incubation time.

**Figure 4:**
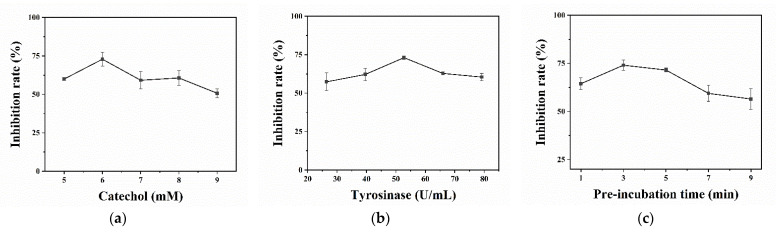
Effects of CA (**a**), concentration of TYR (**b**), and pre-incubation time (**c**) on the inhibition percentage of SBA on TYR. Conditions: 52.75 U/mL of TYR, 5.0–9.0 mM of CA, inhibition and incubation for 5.0 min and 15.0 min at 25 °C, respectively (**a**); 26.375–79.125 U/mL of TYR, 6.0 mM of CA, pre-incubation and incubation for 5.0 min and 15.0 min at 25 °C, respectively (**b**); 52.75 U/mL of TYR, 6.0 mM of CA, pre-incubation and incubation for 1.0–9.0 min and 15.0 min at 25 °C, respectively (**c**) (*n* = 3). The concentrations mentioned above are final concentrations.

On the other hand, to elevate the analytical performance of the developed PGM-based method for the SBA detection, the effects of experimental parameters for the inhibitory activity of SBA on TYR were studied. Figure 4a shows that the effect of the final concentration of CA (5.0–9.0 mM) on the inhibition percentage of SBA on TYR. The inhibition percentage of SBA on TYR reaches a maximum at the final CA concentration of 6.0 mM and then decreases with the increase in the CA concentration. The reason of low inhibition percentage at high CA concentration can be attributed to the competitive inhibition between the SBA and CA. Thus, 6.0 mM was selected as the optimal final CA concentration for the further study. Figure 4b shows that the tested TYR concentrations have little effect on the inhibitory activity of SBA on TYR. The inhibition percentage reaches a maximum value until the final TYR concentration of 52.75 U/mL, which was selected for the following experiment. Finally, the effect of the pre-incubation time (1.0–9.0 min) on the inhibition percentage of TYR by SBA was investigated and the result are shown in Figure 4c. The inhibition percentage of SBA on TYR reaches the maximum at 3.0 min pre-incubation time, and then gradually decreases with further increasing pre-incubation time. The reason may be that SBA can first combine with TYR, prolonging the lag time of catalysis of CA by TYR [46,47]. Thus, 3.0 min was chosen as the optimal pre-incubation time.

### 3.3. Portable Detection of TYR Activity by the PGM-Based Method

Under the optimal conditions, the repeatability of the developed PGM-based method with different glucose test strips was investigated through the relative standard deviation (RSD) of PGM readout. The intra-assay RSD of PGM readout is calculated to be 2.7% using a batch (*n* = 5) of glucose strips, the inter-assay RSD of PGM readout using different batches (*n* = 5) of glucose strips is calculated to be 1.7%. These results suggest that the PGM-based method has satisfactory repeatability.

**Figure 5:**
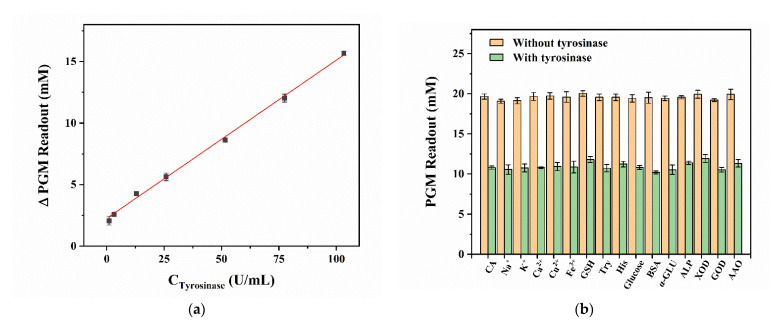
The calibration plot between the TYR activity and the ∆PGM readout (**a**) and effect of various interferences on the ∆PGM readout (**b**). Conditions: 8.0 mM of CA, 1.0–103.3 U/mL of TYR (**a**); 8.0 mM of CA, 52.75 U/mL of TYR, 1.0 mM of Na^+^, K^+^, Ca^2+^, Cu^2+^, and Fe^3+^, 0.5 mM of glucose, 1.0 mM of Try, His, and GSH, 0.33 mg/mL of BSA, 245.7 U/mL of α-GLU, 88.3 U/mL of GOD, 87.7 U/mL of AAO and 100.0 U/mL of ALP and XOD (**b**); incubation at 25 °C for 15.0 min, respectively (*n* = 3). The concentrations mentioned above are final concentrations.

Then, different concentrations of TYR were determined by the developed PGM-based method to obtain a calibration plot. As shown in Figure 5a, the ∆PGM readout increases and the color of the mixture solution gets browner gradually with an increasing concentration of TYR. The calibration plot describes a linear relationship between the ΔPGM readout and concentration of TYR in the range of 1.0–103.3 U/mL with the regression equation of ΔPGM readout = 0.1277 × C_TYR_ + 2.3375 (R^2^ = 0.9958). The limit of quantification (LOQ) for TYR is about 1.0 U/mL, which is comparable to the sensitivity of other TYR activity determination methods, and the liner range is slightly wider than that of some high-sensitive analytical methods (Table S2). In particular, this method requires only a conventional PGM, which provides an easy and portable way for unskilled personnel to monitor TYR activity.

Some potential interferences in the human serum sample were investigated to confirm the selectivity and interferences of the proposed PGM-based method for detecting TYR activity, including metal ions (1.0 mM of Na^+^, K^+^, Ca^2+^, Cu^2+^, and Fe^3+^), biological small molecules (0.5 mM of glucose, 1.0 mM of Try, His, and GSH), enzymes (245.7 U/mL of α-GLU, 88.3 U/mL of GOD, 87.7 U/mL of AAO, 100.0 U/mL of ALP and XOD) and proteins (0.33 mg/mL of BSA). As shown in Figure 5b, the ∆PGM readout variation is no more than 0.7 mM in the presence of potential interferences. Among them, AAO is also a copper-containing oxidase, which has no significant influence on the detection of TYR activity. These results indicate a high selectivity and specificity of the developed method for monitoring TYR activity.

To further investigate the accuracy and practicability of the proposed method for the determination of TYR activity, the spiked recovery test in normal human serum was carried out. The human serum sample was finally diluted 600 times for analysis. The results are summarized in Table S3, and the recoveries of TYR spiked with three different concentrations in the human serum sample (final TYR concentrations of 26.38, 52.75, and 79.13 U/mL) are from 97.9% to 104.8% and the RSDs are between 0.5% and 2.7%, which prove the relatively high accuracy and feasibility of the PGM-based method for the detection of TYR activity in complex biological sample.

### 3.4. Portable Detection of SBA by the PGM-Based Method

**Figure 6:**
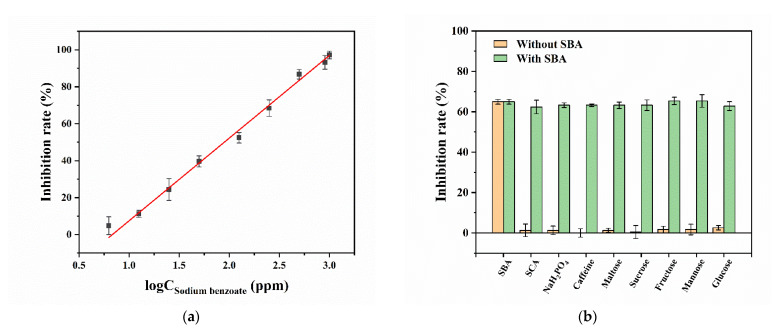
The calibration plot between the logarithm concentration of SBA and the inhibition percent of SBA on TYR (**a**) and effect of various interferences on the inhibition percentage (**b**). Conditions: 52.75 U/mL of TYR, 6.0 mM of CA, 6.25–1000 ppm of SBA (**a**); 200 ppm of SBA, SCA, and caffeine, 2500 ppm of Na_2_HPO_4_, 1000 ppm of maltose, sucrose, fructose, mannose, and glucose (**b**); pre-incubation and incubation for 3.0 min and 15.0 min at 25 °C, respectively (*n* = 3). The concentrations mentioned above are final concentrations.

According to the Equation (3), the *Z’* factor is calculated to be 0.90 (*n* = 8), which indicates that the developed method for the detection of SBA based on the inhibition of SBA on TYR is precise and steady. The Figure 6a shows that the inhibition percentage of SBA on TYR increases gradually with the increase in the logarithm concentration of SBA. The calibration plot was obtained, and the regression equation is inhibition percentage (%) = 44.7379 × logC_SBA_ + 37.2441 (R^2^ = 0.9948) with the SBA concentrations of 6.25–1000 ppm. The LOQ for SBA is about 6.25 ppm, which is not only far lower than the applicable maximum concentration of SBA in carbonated beverages (200 ppm) specified by the CFDA [9], but also lower than some other reported SBA or BA detection methods (Table S4). More importantly, this method does not require any modification to the PGM or materials, and only requires simple reactions to achieve a comparable sensitivity and a wide liner range (6.25–1000 ppm).

To explore the selectivity and interferences of the PGM-based method for the detection of SBA, several potential interferences existed in the three carbonated beverages’ (Cola, Sprite, and Fanta) formulations, including 200 ppm of SCA and caffeine, 2500 ppm of Na_2_HPO_4_, and saccharides (1000 ppm of maltose, sucrose, fructose, mannose, and glucose), were investigated. The results in Figure 6b show that the inhibition percentages of TYR by SBA have no significant (or negligible) effect among these interference solutions with or without the addition of SBA, indicating a good selectivity and specificity of the developed method for the determination of SBA.

In addition, the contents of SBA in Cola, Sprite, and Fanta were determined. The three samples were diluted before analysis, 36 times for Cola and 12 times for Sprite and Fanta, and their background readouts were subtracted in the following analysis (Table S5). As shown in Table 1, the experimental results show that SBA is not present in Cola or its concentration is lower than the LOQ, but the concentrations of SBA detected in Sprite and Fanta are 151.5 ± 9.3 ppm and 179.5 ± 9.4 ppm, respectively. The recoveries of SBA in three different concentrations (final SBA concentrations of 50, 100, and 200 ppm) of the three real samples are in the range of 91.6–106.8%. Moreover, the results measured by the developed PGM-based method was compared with that of HPLC analysis. Figure S4 shows the chromatograms of 10 ppm SBA and three real samples (Cola, Sprite, and Fanta) after diluting 12, 15, and 20 times by 10.0 mM PBS (pH = 6), respectively. It is demonstrated (Table 1) that the SBA concentrations and recoveries determined by the PGM-based method are in good agreement with those obtained by the HPLC method.

## 4. Conclusions

In this study, the PGM was successfully used as a rapid and user-friendly CA-meter based on the reduction of the mediator K_3_[Fe(CN)_6_] to K_4_[Fe(CN)_6_] in the glucose test strip. Subsequently, a simple, convenient, and low-cost PGM-based method for detection of TYR activity and its inhibitor SBA was successfully established based on the catalytic effect of TYR on CA for the first time. This method makes the analysis of TYR activity and SBA as simple as the detection of blood glucose by the PGM, which does not require expensive and bulky instruments and professional operators, greatly reducing the cost. Moreover, the PGM-based method presents a low LOQ, a wide linear range, a high accuracy, and satisfactory selectivity and recovery in real sample analysis. This study offers a promising way to broaden the applications of PGMs in non-glucose targets analysis and POCT in the resource-limited area.

## Figures and Tables

**Table 1:** Recovery studies of SBA in the three carbonate beverages analyzed by the PGM-based and HPLC methods.

Samples	Spiked (ppm)	PGM-Based Method		HPLC Method	
		Found (ppm)	Recovery (%)	Found (ppm)	Recovery (%)
Cola	0	0 ^a^	-	0 ^a^	-
	50	46.2 ± 2.4	92.3	47.9 ± 2.1	95.9
	100	95.1 ± 2.9	95.1	98.4 ± 1.4	98.4
	200	213.5 ± 11.1	106.8	202.6 ± 8.4	101.3
Sprite	0	151.5 ± 9.3	-	156.5 ± 5.1	-
	50	52.2 ± 2.0	104.4	46.9 ± 0.2	93.7
	100	95.7 ± 8.7	95.7	98.5 ± 2.5	98.5
	200	183.3 ± 5.8	91.6	195.8 ± 1.4	97.9
Fanta	0	179.5 ± 9.4	-	183.1 ± 0.3	-
	50	47.3 ± 5.0	94.7	51.7 ± 0.4	103.4
	100	105.5 ± 9.4	105.5	100.0 ± 4.6	100.0
	200	212.4 ± 6.8	106.2	205.3 ± 2.2	102.7

^a^ There is no SBA or its content is lower than the quantification or detection limit of the methods.

## Data Availability

Not applicable.

## Supplementary Materials

The following supporting information can be downloaded at: https://www.mdpi.com/2079-6374/12/12/1084/s1, Figure S1: the color change of K_3_[Fe(CN)_6_] solutions before (a) and 30 s after (c) addition of catechol (CA) (b); Figure S2: the calibration plot for CA determined by PGM (*n* = 3); Figure S3: effect of sodium benzoate (SBA) concentration with different incubation time on the PGM readout of CA (*n* = 3). Conditions: 15.0 mM of CA, 0–2000 ppm of SBA, incubation time of 5.0, 10.0, and 15.0 min, respectively. All the concentrations are final concentration; Figure S4: HPLC chromatograms of SBA (10 ppm) and three carbonated beverages (Cola, Sprite, and Fanta) diluted 12, 15, and 20 times, respectively. 1, SBA; Table S1: some reported tyrosine-based biosensors for the TYR determination; Table S2: comparisons of the developed PGM-based method with the reported methods for the TYR determination; Table S3: recovery studies of TYR in normal human serum detected by the PGM-based method; Table S4: comparisons of the developed PGM-based method with reported methods for the SBA determination; Table S5: the PGM readout of the three beverages spiked with SBA. References [20,39,40,41,48,49,50,51,52,53,54,55,56] are citied in the Supplementary Materials.

## Abbreviations

AAO, ascorbate oxidase; ALP, alkaline phosphatase; BA, benzoic acid; BSA, bovine serum albumin; CA, catechol; CFDA, China Food and Drug Administration; FT-IR, Fourier-transform infrared; GOD, glucose oxidase; GSH, glutathione; His, L-histidine; HPLC, high performance liquid chromatography; PBS, phosphate buffer solution; PGM, personal glucose meter; POCT, point-of-care testing; SBA, sodium benzoate; SCA, trisodium citrate dehydrate; Try, L-tryptophan; TYR, tyrosinase; XOD, xanthine oxidase.

## References

[1] Surekha M., Reddy S.M. (2014). Preservatives|Classification and Properties. Encyclopedia of Food Microbiology.

[2] Del Olmo A., Calzada J., Nunez M. (2017). Benzoic acid and its derivatives as naturally occurring compounds in foods and as additives: Uses, exposure, and controversy. Crit. Rev. Food Sci. Nutr..

[3] Chen H., Brashears M.M., Zhong Q. (2022). Sodium benzoate and sodium bisulfate as preservatives in apple juice and alternative sanitizers for washing cherry tomatoes. Int. J. Food Microbiol..

[4] Nair B. (2001). Final report on the safety assessment of benzyl alcohol, benzoic acid, and sodium benzoate. Int. J. Toxicol..

[5] Piper J.D., Piper P.W. (2017). Benzoate and sorbate salts: A systematic review of the potential hazards of these invaluable preservatives and the expanding spectrum of clinical uses for sodium benzoate. Compr. Rev. Food Sci. Food Saf..

[6] Praphanphoj V., Boyadjiev S.A., Waber L.J., Brusilow S.W., Geraghty M.T. (2000). Three cases of intravenous sodium benzoate and sodium phenylacetate toxicity occurring in the treatment of acute hyperammonaemia. J. Inherit. Metab. Dis..

[7] Qi P., Hong H., Liang X.Y., Liu D.H. (2009). Assessment of benzoic acid levels in milk in China. Food Control..

[8] Azuma S.L., Quartey N.K.A., Ofosu I.W. (2020). Sodium benzoate in non-alcoholic carbonated (soft) drinks: Exposure and health risks. Sci. Afr..

[9] Tan Y., Xie Y., Qiao X., Bai S. (2015). The China Food and Drug Administration (CFDA). Approaching China’s Pharmaceutical Market.

[10] Wei R., Li W., Yang L., Jiang Y., Xie T. (2011). Online preconcentration in capillary electrophoresis with contactless conductivity detection for sensitive determination of sorbic and benzoic acids in soy sauce. Talanta.

[11] Hsu S.H., Hu C.C., Chiu T.C. (2014). Online dynamic pH junction-sweeping for the determination of benzoic and sorbic acids in food products by capillary electrophoresis. Anal. Bioanal. Chem..

[12] Saad B., Bari M.F., Saleh M.I., Ahmad K., Talib M.K. (2005). Simultaneous determination of preservatives (benzoic acid, sorbic acid, methylparaben and propylparaben) in foodstuffs using high-performance liquid chromatography. J. Chromatogr. A.

[13] Pylypiw H.M., Grether M.T. (2000). Rapid high-performance liquid chromatography method for the analysis of sodium benzoate and potassium sorbate in foods. J. Chromatogr. A.

[14] Goren A.C., Bilsel G., Simsek A., Bilsel M., Akcadag F., Topal K., Ozgen H. (2015). HPLC and LC-MS/MS methods for determination of sodium benzoate and potassium sorbate in food and beverages: Performances of local accredited laboratories via proficiency tests in Turkey. Food Chem..

[15] Üstün Özgür M., Kasapoğlu M. (2019). Development and validation of a simple ultra fast liquid chromatographic method for the simultaneous determination of aspartame, acesulfame-k, caffeine and sodium benzoate in dietic soft drinks. J. Anal. Chem..

[16] Zhang H., Yang H., Liu P., Qin X., Liu G. (2022). Colorimetric quantification of sodium benzoate in food by using d-amino acid oxidase and 2D metal organic framework nanosheets mediated cascade enzyme reactions. Talanta.

[17] Fujiyoshi T., Ikami T., Kikukawa K., Kobayashi M., Takai R., Kozaki D., Yamamoto A. (2018). Direct quantitation of the preservatives benzoic and sorbic acid in processed foods using derivative spectrophotometry combined with micro dialysis. Food Chem..

[18] Liu L., Gao Y., Liu J., Li Y., Yin Z., Zhang Y., Pi F., Sun X. (2021). Sensitive techniques for POCT sensing on the residues of pesticides and veterinary drugs in food. Bull. Environ. Contam. Toxicol..

[19] Liu C.C., Wang Y.N., Fu L.M., Chen K.L. (2018). Microfluidic paper-based chip platform for benzoic acid detection in food. Food Chem..

[20] Ko C.H., Liu C.C., Chen K.H., Sheu F., Fu L.M., Chen S.J. (2021). Microfluidic colorimetric analysis system for sodium benzoate detection in foods. Food Chem..

[21] Ray R., Prabhu A., Prasad D., Garlapati V.K., Aminabhavi T.M., Mani N.K., Simal-Gandara J. (2022). Paper-based microfluidic devices for food adulterants: Cost-effective technological monitoring systems. Food Chem..

[22] Musacchio N., Ciullo I., Scardapane M., Giancaterini A., Pessina L., Maino S., Gaiofatto R., Nicolucci A., Rossi M.C., Self-Care Study G. (2018). Efficacy of self-monitoring blood glucose as a key component of a chronic care model versus usual care in type 2 diabetes patients treated with oral agents: Results of a randomized trial. Acta Diabetol..

[23] Xing X., Yao L., Yan C., Xu Z., Xu J., Liu G., Yao B., Chen W. (2021). Recent progress of personal glucose meters integrated methods in food safety hazards detection. Crit. Rev. Food Sci. Nutr..

[24] Zeng L., Gong J., Rong P., Liu C., Chen J. (2019). A portable and quantitative biosensor for cadmium detection using glucometer as the point-of-use device. Talanta.

[25] Zhang H., Chen G.Y., Qian Z.M., Li W.J., Li C.H., Hu Y.J., Yang F.Q. (2021). A portable personal glucose meter method for enzyme activity detection and inhibitory activity evaluation based on alkaline phosphatase-mediated reaction. Anal. Bioanal. Chem..

[26] Chen G.Y., Zhang H., Yang F.Q. (2021). A simple and portable method for beta-Glucosidase activity assay and its inhibitor screening based on a personal glucose meter. Anal. Chim. Acta.

[27] Nie D., Zhang Z., Guo D., Tang Y., Hu X., Huang Q., Zhao Z., Han Z. (2021). A flexible assay strategy for non-glucose targets based on sulfhydryl-terminated liposomes combined with personal glucometer. Biosens. Bioelectron..

[28] Singh N.K., Ray P., Carlin A.F., Magallanes C., Morgan S.C., Laurent L.C., Aronoff-Spencer E.S., Hall D.A. (2021). Hitting the diagnostic sweet spot: Point-of-care SARS-CoV-2 salivary antigen testing with an off-the-shelf glucometer. Biosens. Bioelectron..

[29] Zhang X., Huang X., Wang Z., Zhang Y., Huang X., Li Z., Daglia M., Xiao J., Shi J., Zou X. (2022). Bioinspired nanozyme enabling glucometer readout for portable monitoring of pesticide under resource-scarce environments. Chem. Eng. J..

[30] Tian T., Zhang H., Yang F.Q. (2022). Ascorbate oxidase enabling glucometer readout for portable detection of hydrogen peroxide. Enzym. Microb. Technol..

[31] Zhang H., Gong Z.M., Li Y., Yang F.Q. (2022). A simple and green method for direct determination of hydrogen peroxide and hypochlorite in household disinfectants based on personal glucose meter. Enzym. Microb. Technol..

[32] Tief K., Hahne M., Schmidt A., Beermann F. (1996). Tyrosinase, the key enzyme in melanin synthesis, is expressed in murine brain. Eur. J. Biochem..

[33] Rolff M., Schottenheim J., Decker H., Tuczek F. (2011). Copper-O_2_ reactivity of tyrosinase models towards external monophenolic substrates: Molecular mechanism and comparison with the enzyme. Chem. Soc. Rev..

[34] Fartas F.M., Abdullah J., Yusof N.A., Sulaiman Y., Saiman M.I. (2017). Biosensor based on tyrosinase immobilized on graphene-decorated gold nanoparticle/chitosan for phenolic detection in aqueous. Sensors.

[35] Cancelliere R., Carbone K., Pagano M., Cacciotti I., Micheli L. (2019). Biochar from brewers’ spent grain: A green and low-cost smart material to modify screen-printed electrodes. Biosensors.

[36] Chang T.S. (2009). An updated review of tyrosinase inhibitors. Int. J. Mol. Sci..

[37] Paisan-Ruiz C., Houlden H. (2010). Common pathogenic pathways in melanoma and Parkinson disease. Neurology.

[38] Fan Y.F., Zhu S.X., Hou F.B., Zhao D.F., Pan Q.S., Xiang Y.W., Qian X.K., Ge G.B., Wang P. (2021). Spectrophotometric assays for sensing tyrosinase activity and their applications. Biosensors.

[39] Ma X., Gao W., Halawa M.I., Lan Y., Li J., Xu G. (2019). Lucigenin fluorescent assay of tyrosinase activity and its inhibitor screening. Sens. Actuators B Chem..

[40] Ren H., Xu T., Liang K., Li J., Fang Y., Li F., Chen Y., Zhang H., Li D., Tang Y. (2022). Self-assembled peptides-modified flexible field-effect transistors for tyrosinase detection. iScience.

[41] Baron R., Zayats M., Willner I. (2005). Dopamine-, L-DOPA-, adrenaline-, and noradrenaline-induced growth of Au nanoparticles: Assays for the detection of neurotransmitters and of tyrosinase activity. Anal. Chem..

[42] Cooksey C.J., Garratt P.J., Land E.J., Ramsden C.A., Riley P.A. (1998). Tyrosinase kinetics: Failure of the auto-activation mechanism of monohydric phenol oxidation by rapid formation of a quinomethane intermediate. Biochem. J..

[43] Menon S., Fleck R.W., Yong G., Strothkamp K.G. (1990). Benzoic acid inhibition of the α, β, and γ Isozymes of *Agaricus bisporus* tyrosinase. Arch. Biochem. Biophys..

[44] Le Caër S., Vigneron G., Renault J.P., Pommeret S. (2006). First coupling between a LINAC and FT-IR spectroscopy: The aqueous ferrocyanide system. Chem. Phys. Lett..

[45] Yu L., Zhang Q., Jin D., Xu Q., Hu X. (2019). A promising voltammetric biosensor based on glutamate dehydrogenase/Fe_3_O_4_/graphene/chitosan nanobiocomposite for sensitive ammonium determination in PM_2.5_. Talanta.

[46] Sánchez-Ferrer Á., Neptuno Rodríguez-López J., García-Cánovas F., García-Carmona F. (1995). Tyrosinase: A comprehensive review of its mechanism. Biochim. Biophys. Acta (BBA) Protein Struct. Mol. Enzymol..

[47] Cabanes J., García-Cánovas F., Lozano J., García-Carmona F. (1987). A kinetic study of the melanization pathway between L-tyrosine and dopachrome. Biochim. Biophys. Acta (BBA) Gen. Subj..

[48] Liu Y.B., Zhang Y., Zhang X.L., Zhang W., Wang X.H., Sun Y., Ma P.Y., Huang Y.B., Song D.Q. (2021). Near-infrared fluorescent probe based on Ag&Mn:ZnInS QDs for tyrosinase activity detection and inhibitor screening. Sens. Actuators B Chem..

[49] Li Y.N., Deng B., Yang S.X., Tian H.Y., Liu Y.G., Sun B.G. (2021). A fluorescent probe for the visible colorimetric detection of tyrosinase. Chemistryselect.

[50] Wu X., Li L., Shi W., Gong Q., Ma H. (2016). Near-infrared fluorescent probe with new recognition moiety for specific detection of tyrosinase activity: Design, synthesis, and application in living cells and zebrafish. Angew. Chem.-Int. Edit..

[51] Shi L., Zhang Z.H., Zhang L.M., Tian Y. (2022). Electrochemical detection of tyrosinase in cell lysates at functionalized nanochannels via amplifying of ionic current response. Electroanalysis.

[52] Cai S., Chen X., Wang L., Xie L., Liu J., Zheng J. (2022). Effective detection of tyrosinase by Keggin-type polyoxometalate-based electrochemical sensor. J. Solid State Electrochem..

[53] D’Amore T., Di Taranto A., Berardi G., Vita V., Iammarino M. (2021). Going green in food analysis: A rapid and accurate method for the determination of sorbic acid and benzoic acid in foods by capillary ion chromatography with conductivity detection. LWT-Food Sci. Technol..

[54] Iammarino M., Taranto A.D. (2013). Development and validation of an ion chromatography method for the simultaneous determination of seven food additives in cheeses. J. Anal. Sci..

[55] Xue L., Chen L., Dong J., Cai L., Wang Y., Chen X. (2020). Dispersive liquid-liquid microextraction coupled with surface enhanced Raman scattering for the rapid detection of sodium benzoate. Talanta.

[56] Hamzah H.H., Yusof N.A., Salleh A.B., Bakar F.A. (2011). An optical test strip for the detection of benzoic acid in food. Sensors.

