# Supplementary material for: A Catechol-Meter Based on Conventional Personal Glucose Meter for Portable Detection of Tyrosinase and Sodium Benzoate
Source: Biosensors (Basel). 2022 Nov 27;12(12):1084. doi: 10.3390/bios12121084 (PMC9776396; doi:10.3390/bios12121084)
Supplement: Supplementary file 1 [file biosensors-12-01084-s001.zip › biosensors-2020609-supplementary.pdf]

Supplementary Information

A catechol-meter based on conventional personal glucose meter for portable detection of tyrosinase and sodium benzoate

Tao Tian [1,2], Wei-Yi Zhang [2], Hang-Yu Zhou [2], Li-Jing Peng [2], Xi Zhou [2], Hao Zhang [1,*] and Feng-Qing Yang [2]

[1] Chongqing Key Laboratory of High Active Traditional Chinese Drug Delivery System, Chongqing Medical and Pharmaceutical College, Chongqing 401331, China

[2] School of Chemistry and Chemical Engineering, Chongqing University, Chongqing 401331, China

* Correspondence: zhanghao199392@163.com

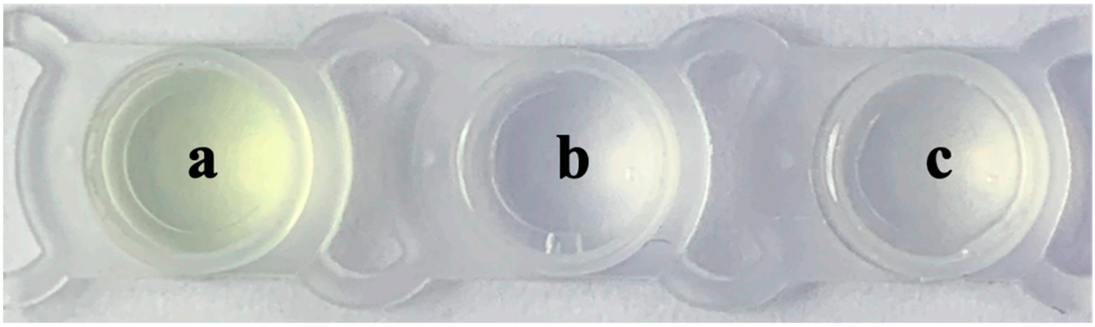

Figure S1. The color change of $K_3[Fe(CN)_6]$ solutions before (**a**) and 30 s after (**c**) addition of catechol (CA) (**b**).

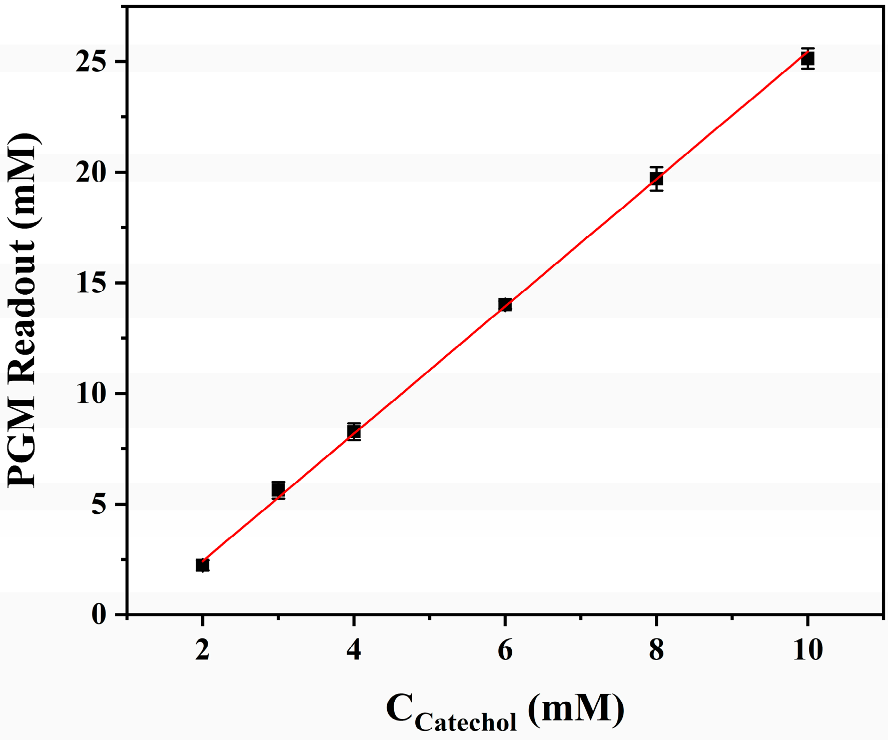

Figure S2. The calibration plot for CA determined by PGM (*n*=3).

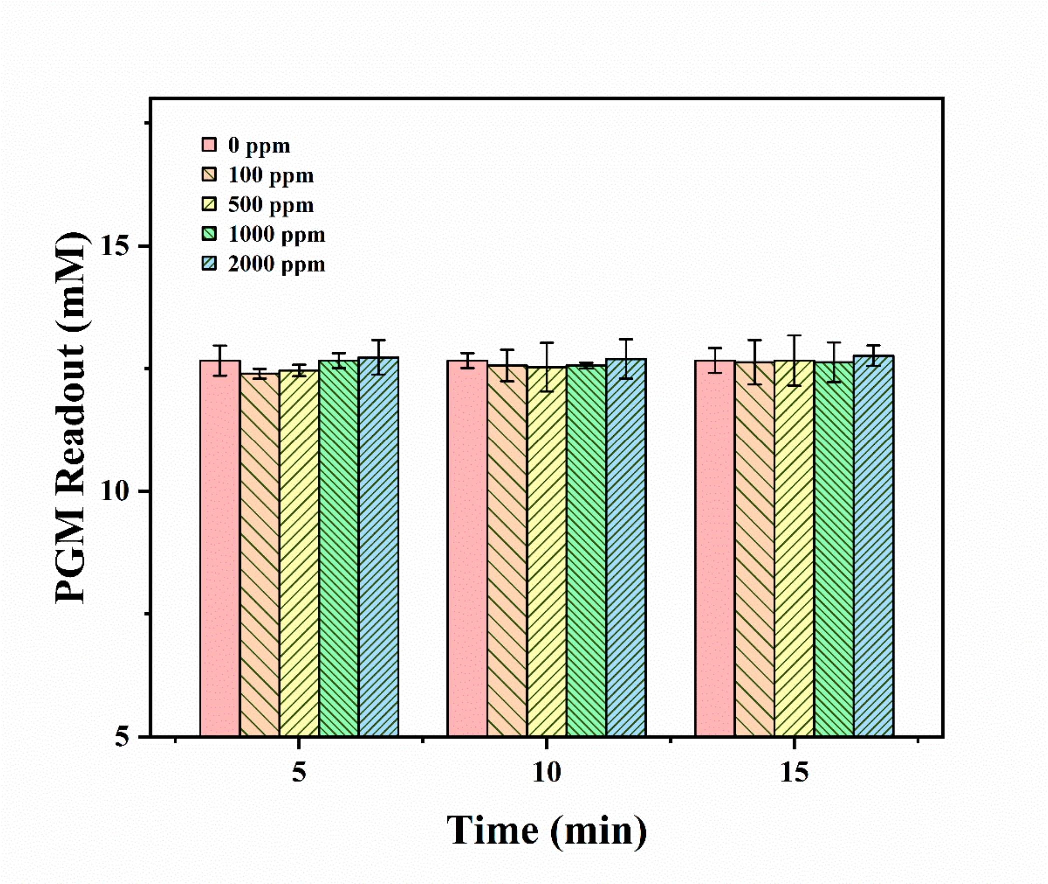

Figure S3. Effect of sodium benzoate (SBA) concentration with different incubation time on the PGM readout of CA (*n*=3). Conditions: 15.0 mM of CA, 0–2000 ppm of SBA, incubation time of 5.0, 10.0, and 15.0 min, respectively. All the concentrations are final concentration.

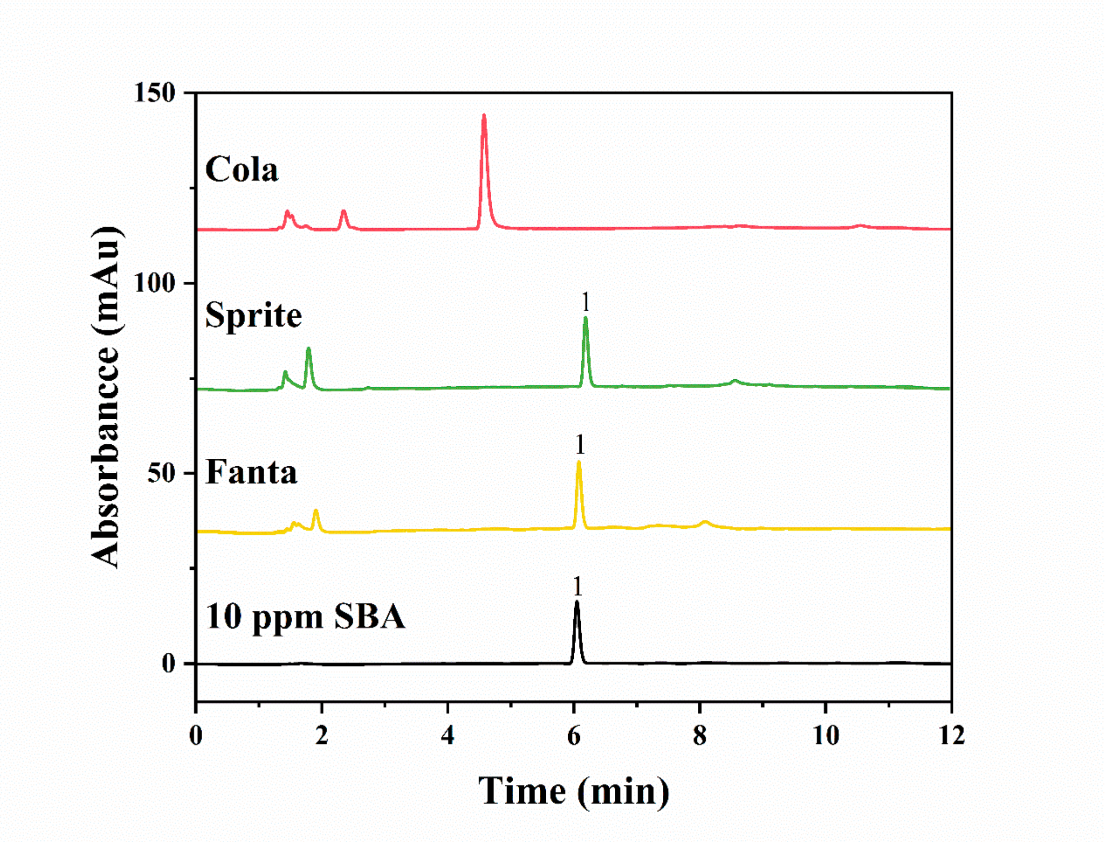

Figure S4. HPLC chromatograms of SBA (10 ppm) and three carbonated beverages (Cola, Sprite, and Fanta) diluted 12, 15, and 20 times, respectively. 1, SBA.

Table S1. Some reported tyrosine-based biosensors for the TYR determination.

Methods	Materials	Line range	LOD	References
Fluorescence	Lucigenin probe	0.67–67.0 U/mL	0.54 U/mL	[1]
Fluorescence	Ag&Mn:ZnInS QDs	0.5–2.5 U/mL	0.09 U/mL	[2]
Electrochemistry	WVFY-modified bio-FETs	10 fM–1 nM	1.9 fM	[3]

QDs: quantum dots; WVFY: tetrapeptide tryptophan–valine–phenylalanine–tyrosine; bio-FETs: field-effect transistor-based biosensors; LOD: limit of detection.

Table S2. Comparisons of the developed PGM-based method with the reported methods for the TYR determination.

Methods	Line range (U·mL^{-1})	LOD / LOQ (U·mL^{-1})	Cost	Operator	References
Colorimetry	–	10 / –	High	Highly trained	[4]
Fluorescence	0–800	5	High	Highly trained	[5]
Fluorescence	0–80	2.76 / –	High	Highly trained	[6]
Electrochemistry	2–50	0.83 / –	High	Highly trained	[7]
Electrochemistry	3.76–27.68	0.52/ –	High	Highly trained	[8]
PGM	1.0–103.3	– / 1.0	Low	Minimally trained	This work

LOD: limit of detection; LOQ: limit of quantitation.

Table S3. Recovery studies of TYR in normal human serum detected by the PGM-based method.

Sample	Spiked tyrosinase (U/mL)	Found tyrosinase (U/mL)	Recovery (%)	RSD (*n* = 3, %)
Normal human serum	0[a]	–	–	–
	26.38	27.64 ±0.74	104.8	2.7
	52.75	51.91 ± 0.98	98.4	1.9
	79.13	77.49 ± 0.37	97.9	0.5

[a] There is no TYR or its content lower than the quantification limit of the PGM-based method.

Table S4. Comparisons of the developed PGM-based method with reported methods for the SBA determination.

Methods	Analytes	Line range (ppm)	LOD / LOQ (ppm)	References
CIC-CD	BA	0.25–20	4.1 / 12.6	[9]
IC-CD	BA	-	16.5 / 54.5	[10]
DLLME-SERS	SBA	10–500	0.56 / –	[11]
Paper-based	BA	100–700	73.6	[12]
MCA system	SBA	500–5000	50 / –	[13]
PGM-based	SBA	6.25–1000	– / 6.25	This work

CIC-CD: Capillary ion chromatography with conductivity detection; IC-CD: Ion chromatography with conductivity detection; DLLME-SERS: Dispersive liquid-liquid microextraction combined with surface enhanced Raman scattering; MCA: Microfluidic colorimetric analysis

Table S5. The PGM readout of the three beverages spiked with SBA.

Spiked (ppm) (*n*=3)	PGM Readout (mM)		
	Cola	Sprite	Fanta
0	12.1 ± 0.20	17.9 ± 0.12	16.6 ± 0.10
50	15.1 ± 0.10	20.4 ± 0.06	18.8 ± 0.15
100	16.2 ± 0.06	21.2 ± 0.15	19.9 ± 0.15
200	17.5 ± 0.10	22.2 ± 0.06	20.9 ± 0.06
Background readout	6.2 ± 0.25	11.0 ± 0.15	9.5 ± 0.36

References

1. Ma, X.G.; Gao, W.Y.; Halawa, M.I.; Lan, Y.X.; Li, J.P.; Xu, G.B. Lucigenin fluorescent assay of tyrosinase activity and its inhibitor screening. *Sensors and Actuators B: Chemical* **2019**, *280*, 41-45. [https://doi.org/10.1016/j.snb.2018.10.044]
2. Liu, Y.B.; Zhang, Y.; Zhang, X.L.; Zhang, W.; Wang, X.H.; Sun, Y.; Ma, P.Y.; Huang, Y.B.; Song, D.Q. Near-infrared fluorescent probe based on Ag&Mn:ZnInS QDs for tyrosinase activity detection and inhibitor screening. *Sensors and Actuators B: Chemical* **2021**, *344*, 130234. [https://doi.org/10.1016/j.snb.2021.130234]
3. Ren, H.; Xu, T.; Liang, K.; Li, J.; Fang, Y.; Li, F.; Chen, Y.; Zhang, H.; Li, D.; Tang, Y.; et al. Self-assembled peptides-modified flexible field-effect transistors for tyrosinase detection. *iScience* **2022**, *25*, 103673. [https://doi.org/10.1016/j.isci.2021.103673]
4. Baron, R.; Zayats, M.; Willner, I. Dopamine-, L-DOPA-, adrenaline-, and noradrenaline-induced growth of Au nanoparticles: assays for the detection of neurotransmitters and of tyrosinase activity. *Analytical Chemistry* **2005**, *77*, 1566-1571. [https://doi.org/10.1021/ac048691v]
5. Li, Y.N.; Deng, B.; Yang, S.X.; Tian, H.Y.; Liu, Y.G.; Sun, B.G. A fluorescent probe for the visible colorimetric detection of tyrosinase. *Chemistryselect* **2021**, *6*, 9046-9051. [https://doi.org/10.1002/slct.202102473]
6. Wu, X.; Li, L.; Shi, W.; Gong, Q.; Ma, H. Near-infrared fluorescent probe with new recognition moiety for specific detection of tyrosinase activity: Design, synthesis, and application in living cells and zebrafish. *Angewandte Chemie-International Edition* **2016**, *55*, 14728-14732. [https://doi.org/10.1002/anie.201609895]
7. Shi, L.; Zhang, Z.H.; Zhang, L.M.; Tian, Y. Electrochemical detection of tyrosinase in cell lysates at functionalized nanochannels via amplifying of ionic current response. *Electroanalysis* **2022**, *34*, 1021-1026. [https://doi.org/10.1002/elan.202100358]
8. Cai, S.; Chen, X.; Wang, L.; Xie, L.; Liu, J.; Zheng, J. Effective detection of tyrosinase by Keggin-type polyoxometalate-based electrochemical sensor. *Journal of Solid State Electrochemistry* **2022**, *26*, 419-429. [https://doi.org/10.1007/s10008-021-05085-8]
9. D'Amore, T.; Di Taranto, A.; Berardi, G.; Vita, V.; Iammarino, M. Going green in food analysis: A rapid and accurate method for the determination of sorbic acid and benzoic acid in foods by capillary ion chromatography with conductivity detection. *Lwt-Food Science and Technology* **2021**, *141*, 110841. [https://doi.org/10.1016/j.lwt.2020.110841]
10. Iammarino, M.; Taranto, A.D. Development and validation of an ion chromatography method for the simultaneous determination of seven food additives in cheeses. *Journal of Analytical Sciences* **2013**, *3*, 30-37. [https://doi.org/10.4236/jasmi.2013.33A005]
11. Xue, L.; Chen, L.; Dong, J.; Cai, L.; Wang, Y.; Chen, X. Dispersive liquid-liquid microextraction coupled with surface enhanced Raman scattering for the rapid detection of sodium benzoate. *Talanta* **2020**, *208*, 120360. [https://doi.org/10.1016/j.talanta.2019.120360]

12. Hamzah, H.H.; Yusof, N.A.; Salleh, A.B.; Bakar, F.A. An optical test strip for the detection of benzoic acid in food. *Sensors (Basel)* **2011**, *11*, 7302-7313. [https://doi.org/10.3390/s110807302]
13. Ko, C.H.; Liu, C.C.; Chen, K.H.; Sheu, F.; Fu, L.M.; Chen, S.J. Microfluidic colorimetric analysis system for sodium benzoate detection in foods. *Food Chemistry* **2021**, *345*, 128773. [https://doi.org/10.1016/j.foodchem.2020.128773]
